# Study on the effectiveness and safety of balance instrument training on balance and lower limb motor function in patients with ischemic stroke hemiplegia

**DOI:** 10.3389/fresc.2026.1806869

**Published:** 2026-05-13

**Authors:** Zhimin Chen, Huafang Chen, Junjie Wang, Haodong Lu

**Affiliations:** 1 Rehabilitation Department, Ningbo Municipal Hospital of Traditional Chinese Medicine (TCM), Affiliated Hospital of Zhejiang Chinese Medical University, Ningbo, China; 2 Clinnical Trial Office, First Affiliated Hospital of Wenzhou Medical University, Wenzhou, China

**Keywords:** balance function, balance instrument training, hemiplegia, ischemic stroke, lower limb motor controlfunction, rehabilitation

## Abstract

**Objective:**

This study aimed to investigate the efficacy of a balance instrument (Pro-Kin system) on balance function and lower limb motor function in patients with ischemic stroke hemiplegia.

**Methods:**

Sixty patients with ischemic stroke hemiplegia were randomly assigned to either an experimental group (*n* = 30) or a control group (*n* = 30). Both groups received conventional rehabilitation therapy. The experimental group underwent additional balance training using the Pro-Kin balance measure and training system. Balance function and lower limb motor function were assessed before and after a 6-week intervention using four clinical scales and six parameters derived from the balance instrument.

**Results:**

After adjusting for baseline va lues using an analysis of covariance (ANCOVA), the experimental group demonstrated significantly greater improvements than the control group in all four clinical scales and six balance instrument parameters. Clinical scores: Berg Balance Scale (BBS *F* = 68.42, *P* < 0.001), Fugl-Meyer Assessment for Lower Extremity (FMA-LE *F* = 285.64, *P* < 0.001), Timed Up and Go Test (TUGT *F* = 121.17, *P* < 0.001), and affected-side single-leg stance time (TALS *F* = 27.86, *P* < 0.001) are significantly better (all *P* < 0.05) in the experimental group.The six balance instrument parameters: center of pressure (COP) sway amplitude in the mediolateral (*F* = 24.32, *P* < 0.001) and anteroposterior directions (*F* = 38.74, *P* < 0.001), average sway velocity in the mediolateral (*F* = 59.40, *P* < 0.001) and anteroposterior directions (*F* = 158.64, *P* < 0.001), Motion length of COP in 30s (*F* = 209.03, *P* < 0.001), and Motion ellipse area (*F* = 255.05, *P* < 0.001) are significantly better (all *P* < 0.05) in the experimental group.

**Conclusion:**

Balance instrument training with the Pro-Kin system can effectively improve balance function and lower limb motor function in patients with ischemic stroke hemiplegia, with superior outcomes compared to conventional rehabilitation training alone.

## Introduction

Stroke remains one of the leading causes of mortality and long-term disability worldwide (1). In stroke patients, damage to higher cortical centers disrupts normal synaptic connections and impairs control over lower motor centers, leading to symptoms such as balance dysfunction, impaired inter-muscular coordination, and loss of muscle strength (2). Statistical analyses indicate that approximately 83% of first-episode stroke patients experience balance dysfunction (3). Post-stroke sensory impairments and limb motor dysfunction are common contributors to diminished balance, reduced lower limb motor function, and an increased risk of falls. Notably, about 70% of stroke patients experience a fall within six months after hospital discharge (4). While early intravenous thrombolysis achieves vascular recanalization in approximately 60% of acute ischemic stroke cases, a considerable proportion of patients do not derive optimal benefit from this treatment due to various factors, including presentation outside the therapeutic time window, contraindications to thrombolytic agents, unsuccessful recanalization, or hemorrhagic transformation complications (5). Therefore, rehabilitation training is often essential for restoring motor function in ischemic stroke survivors.

Neural plasticity in the brain can be promoted through the repetitive practice of specific tasks, necessitating that patients engage in sufficient, targeted repetitive activities. Although conventional balance training is beneficial for improving balance function, it is often monotonous and may fail to sustain patient engagement over the required duration of therapy. Furthermore, conventional approaches impose a high physical burden on occupational therapists, potentially limiting treatment intensity and consistency. Technology-assisted rehabilitation has emerged as a promising alternative that may address these limitations by providing real-time biofeedback, gamified exercise environments, and objective performance monitoring, thereby enhancing patient motivation and therapeutic precision (6, 7). The Pro-Kin balance system, in particular, offers visual feedback of center of pressure displacement and enables customized, progressive training protocols. However, despite its theoretical advantages, rigorous evidence regarding its efficacy compared to conventional rehabilitation in ischemic stroke patients with hemiplegia remains limited. Therefore, this study aimed to compare the effects of technology-assisted balance instrument training using the Pro-Kin system with those of routine rehabilitation training on balance function and lower limb motor function in stroke patients, aiming to identify a more convenient, engaging, and effective rehabilitation method.

## Materials and methods

### Study design and participants

**Figure 1:**
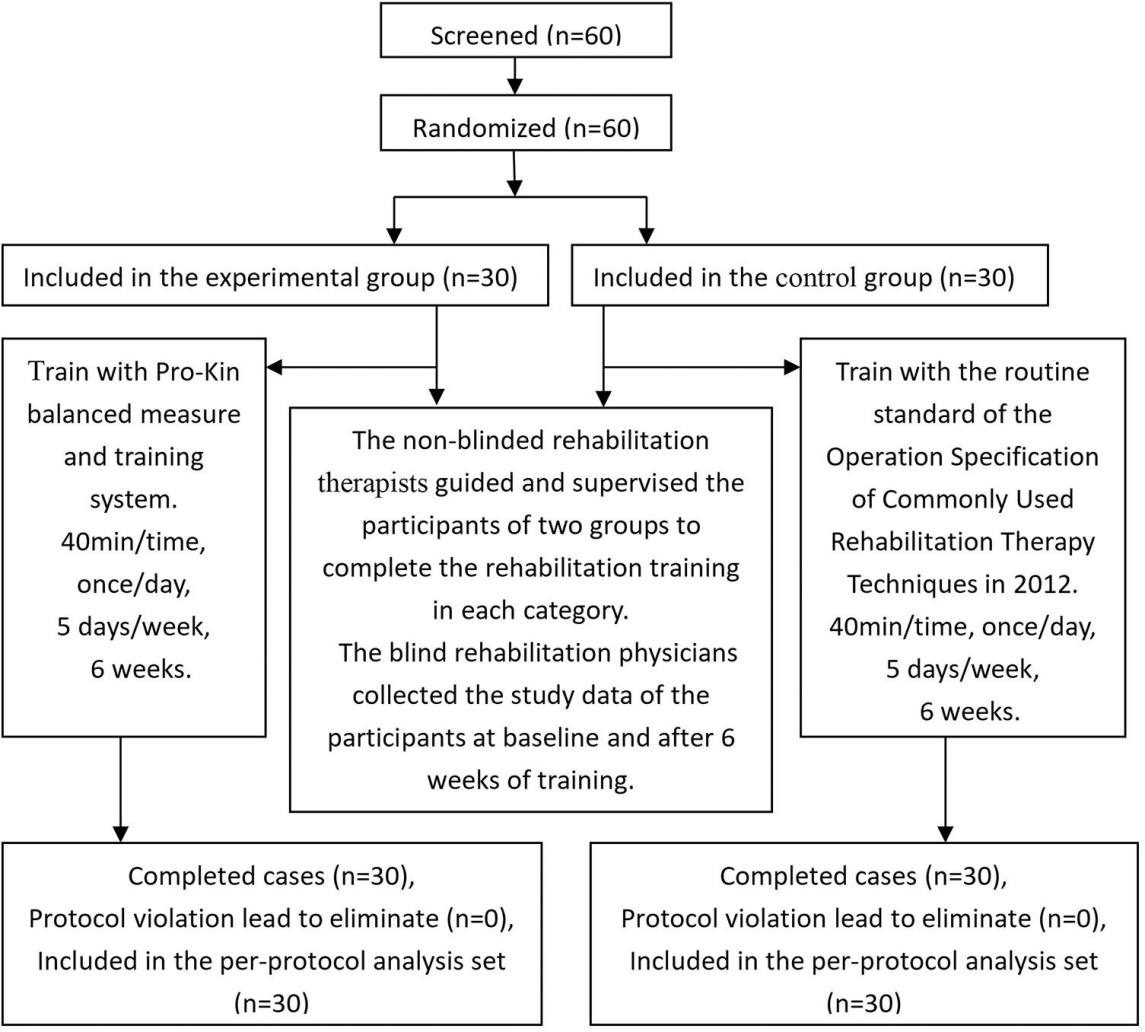
Random grouping of research participants and flowcharts.

This single-center, randomized controlled trial was conducted in the Department of Rehabilitation at Ningbo Municipal Hospital of Traditional Chinese Medicine (TCM), an affiliated hospital of Zhejiang Chinese Medicine University. A total of 60 patients with ischemic stroke hemiplegia were enrolled between April 2023 and June 2025 (Figure 1).

### Ethical approval

The study protocol was approved by the Medical Ethics Committee of Ningbo Municipal Hospital of TCM (Approval No. 2023-01-01, dated 10 January 2023) and was conducted in accordance with the Declaration of Helsinki. All participants provided written informed consent after being fully informed of the study procedures. Their voluntary participation was emphasized.

Inclusion Criteria

1. Diagnosis of ischemic stroke with balance dysfunction and hemiplegia confirmed by CT or MRI and established by a neurologist or rehabilitation physician according to the diagnostic criteria of the Chinese Classification of Cerebrovascular Diseases (2015).
2. Medically stable condition, with no other comorbidities (e.g., progressive neurological worsening, severe dizziness) that would interfere with limb function training or balance assessment.
3. Clear consciousness, with cognitive function adequate to understand instructions and cooperate with assessment and training, operationalized as a Mini-Mental State Examination (MMSE) score ≥24 (8), and absence of significant speech dysfunction that would preclude communication.
4. First-time ischemic stroke, with a disease course of 15–180 days (subacute to convalescent stage).
5. Age between 35 and 80 years.
6. Standing balance function rated at level 1 or higher, ability to stand with assistance for 20 min, muscle strength of the hemiplegic lower limb graded 3 or higher on the Medical Research Council (MRC) scale, spasticity graded ≤1+ on the Modified Ashworth Scale (MAS) to ensure sufficient range of motion for training, and Brunnstrom recovery stage of the lower extremity between III and V.
7. Presence of at least partial proprioceptive sensation in the affected lower limb, defined as the ability to correctly identify direction of passive movement at the ankle or knee joint in ≥3 out of 5 trials.

Exclusion Criteria

Transient ischemic attack or reversible ischemic neurological deficit.
History of two or more strokes.
Severe anxiety, depression, or other psychiatric disorders hindering normal intervention or assessment.
Comorbidities affecting lower limb motor function (e.g., fracture, amputation, severe arthritis), and severe spasticity (MAS ≥ 2).
5. Diseases of severe cardiac, hepatic, pulmonary, renal, endocrine, hematopoietic system, or malignancy.
Poor compliance as judged by the investigators.

### Intervention methods

All participants in both groups received conventional pharmacological treatment for ischemic stroke as prescribed by a rehabilitation physician, in accordance with clinical guidelines.

Control Group: Participants received routine rehabilitation treatment based on the Operation Specification of Commonly Used Rehabilitation Therapy Techniques issued by the Chinese Ministry of Health (2012 edition) (9). Depending on individual toleranceand under the supervision of a licensed physical therapist, training including: (1) passive range of motion exercises for the hemiplegic lower limb (10 min); (2) anti-spasticity positioning and stretching exercises (5 min); (3) static sitting balance training (5 min); (4) dynamic sitting balance training with weight shifting (5 min); (5) parallel bar standing training with gradual reduction of upper limb support (5 min); (6) weight-shifting training in standing (5 min); and (7) gait preparation and walking training (5 min). Sessions lasted 40 min, once daily, 5 days per week, for 6 weeks.Progression was based on individual patient performance, with increasing difficulty (e.g., reduced support, added perturbations) as tolerated. To ensure standardization and consistency, all therapy sessions were delivered by the same team of three licensed therapists who met regularly to discuss and align their treatment approaches based on the patient's individual tolerance and functional level.

Experimental Group: In addition to the conventional therapy received by the control group, participants underwent training using the Pro-Kin balance measure and training system (PKB-MANOP-07/PROKIN Systems 212-252, TecnoBody, Italy; Registration No. 20192191991). Before training, a proprioceptive evaluation was conducted using the Pro-Kin system's assessment protocol. Participants stood in a standardized position while the system recorded measurement results across eight areas (S1–S8), identifying restricted zones.and quantifying weight-bearing asymmetry and proprioceptive deficits. Training programs were then customized based on these results, with particular attention to the participant's baseline proprioceptive accuracy (typically 60%–80% in the study sample). A therapist guided participants to perform game-based exercises by shifting their body weight to control on-screen targets. The training included:

1. Static Balance Training in Standing: Participants maintained a static standing posture while observing real-time feedback of their weight-bearing distribution and center of pressure (COP) trajectory on a monitor. Exercises included COP maintenance, and anterior-posterior and mediolateral weight shifting, progressing to single-leg stance.
2. Dynamic Balance Training in Standing: Participants moved their feet in different directions to shift weight, following visual cues on the monitor to complete selected programs aimed at improving upright dynamic balance.

Training was supervised by a qualified therapist. Sessions were stopped immediately if dizziness or discomfort occurred. The training schedule was identical to the control group: 40 min/day, 5 days/week for 6 weeks.

### Outcome measures

Balance function and lower limb motor function were assessed at baseline and after the 6-week intervention.

Primary Outcomes (Clinical Scales)

1. Berg Balance Scale (BBS): A 14-item scale assessing static and dynamic balance.
2. Fugl-Meyer Assessment for Lower Extremity (FMA-LE): Evaluates motor function recovery of the lower limb (10).
3. Affected-side Single-Leg Stance Time (TALS): Records the maximum time a patient can stand on the affected leg.
4. Timed Up and Go Test (TUGT): The Timed Up and Go Test (TUGT) was included as it is a well-validated, clinically feasible measure that integrates multiple functional components—sit-to-stand transfer, walking, turning, and gait initiation/termination—that are essential for community ambulation. Previous research has established TUGT as a reliable and responsive measure for assessing functional mobility and predicting fall risk in stroke populations (11, 12), making it particularly suitable for evaluating ecologically valid outcomes of balance training.

Instrument-Derived Parameters (Pro-Kin System): The Pro-Kin system was used to assess six center of pressure (COP)-related parameters, which are widely recognized as objective, continuous measures of postural stability (13) and were considered co-primary outcomes due to their high measurement precision:

1. Standard deviation of COP in the mediolateral (ML) and anteroposterior (AP) directions, indicating average sway amplitude. Larger values indicate poorer balance.
2. Average sway velocity in the ML and AP directions. Higher velocities indicate greater postural instability and altered muscle tone regulation.
3. Total sway path length: The total trajectory length of COP movement during a 30-s test, reflecting the overall magnitude of body sway.
4. 95% confidence ellipse area: The area encompassing 95% of COP data points, reflecting the degree of postural sway and stability. Larger values for parameters 5 and 6 indicate worse balance ability.

### Sample size, randomization, and blinding

A simple randomization sequence was generated using Microsoft Excel by a researcher (H.C.) who was not involved in participant recruitment or assessment. Participants were assigned sequential numbers corresponding to sealed, opaque envelopes containing group allocation (experimental or control). These envelopes were prepared and sequentially numbered by a research assistant not involved in the trial. Upon enrollment of a participant, the enrolling therapist opened the next numbered envelope in the presence of the participant to reveal the assignment. Participants and treating therapists were aware of the group allocation due to the nature of the intervention. However, the outcome assessor was blinded to group assignment and was not involved in the intervention delivery.

### Sample size estimation

Sample size was calculated *a priori* based on the primary outcome measure (Berg Balance Scale). Based on pilot data from our preliminary study (*n* = 10 per group), we anticipated a mean difference of 5.0 points in BBS improvement between groups, with a standard deviation of 4.5 points. Using a two-tailed independent samples *t*-test with *α* = 0.05 and power (1 − *β*) = 0.80, the required sample size was calculated as 26 participants per group. Accounting for an anticipated dropout rate of approximately 15%, we aimed to recruit 30 participants per group (total *N* = 60). Sample size calculation was performed using G Power software (version 3.1.9.7; Heinrich Heine University, Dusseldorf, Germany).

### Statistical analysis

All analyses were performed on a per-protocol basis, as all 60 enrolled participants completed the full 6-week intervention and assessments with no dropouts. Data were analyzed using SPSS software (version 23.0). The Shapiro–Wilk test was used to check normality. Continuous data with normal distribution are presented as mean ± standard deviation (SD) and were analyzed using paired *t*-tests (within-group) and independent sample *t*-tests (between-group). Non-normally distributed data are presented as median (interquartile range, IQR) and were analyzed using the Wilcoxon signed-rank test (within-group) and Mann–Whitney *U* test (between-group). Categorical data are presented as counts and were compared using the chi-square test. Statistical significance was set at *P* < 0.05.To account for potential baseline differences and provide a more robust estimate of the treatment effect, an analysis of covariance (ANCOVA) was employed for between-group comparisons of post-intervention outcomes. In each ANCOVA model, the post-intervention value was entered as the dependent variable, the group assignment (experimental or control) as the fixed factor, and the corresponding baseline value as the covariate. Normality of residuals and homogeneity of regression slopes were checked prior to analysis and the assumptions were met.

## Results

All 60 enrolled participants completed the 6-week study protocol.Good compliance was attributed to consistent diagnosis, therapeutic guidance from therapists, and appropriate encouragement.

Demographic and clinical characteristics, including sex, age, body mass index (BMI), affected side, and disease duration, were comparable between the two groups at baseline (all *P* > 0.05, Table 1). Furthermore, no significant differences were observed in any of the six COP parameters or the four clinical scale scores at baseline (all *P* > 0.05, Table 2).

**Table 1:** Baseline demographic and clinical characteristics of the participants.

Group	*N*	Sex		Age (years) (mean ± SD)	BMI (kg/m^2^) (mean ± SD)	Disease course	Paralysis side	
		Male	Female			Days	Left	Right
Experimental	30	18	12	59.0 ± 16.2	25.8 ± 5.35	28 ± 13.7	16	14
Control	30	17	13	58.9 ± 16.9	26.0 ± 5.20	29 ± 14.1	15	15

BMI (Body Mass Index): weight divided by height squared; SD, standard deviation.

**Table 2:** Comparison of baseline data between the two groups.

Variable (Units)	Experimental group (*n* = 30)	Control group (*n* = 30)	*p*-value
Berg Balance Scale (score)	25.00 (22.00,28.00)	26.00 (22.00,28.00)	>0.05
Fugl-Meyer Assessment-LE (score)	7.00 (6.00, 7.00)	7.00 (6.00, 8.00)	>0.05
Timed Up and Go Test (s)	19.00 (18.00, 21.00)	19.00 (17.00, 21.00)	>0.05
Affected-Leg Stance Time (s)	1.88 (0.889, 3.50)	2.10 (1.00, 3.00)	>0.05
COP ML Sway Amplitude (mm)	5.83 (4.49, 6.94)	5.70 ± 1.32	>0.05
COP AP Sway Amplitude (mm)	6.21 (5.00, 7.38)	6.13 ± 1.25	>0.05
ML Sway Velocity (mm/s)	8.08 (7.09, 10.09)	8.31 (6.47, 10.26)	>0.05
AP Sway Velocity (mm/s)	11.42 ± 1.77	11.26 (9.50, 13.59)	>0.05
Total Sway Path Length (mm)	547.87 ± 69.93	542.32 (489.60, 602.88)	>0.05
Motion ellipse area (mm^2^)	552.15 ± 74.18	539.43 ± 76.61	>0.05

AP, anteroposterior; COP, Center of Pressure; LE, lower extremity; ML, mediolateral. Data presented as mean ± SD or median (IQR). Denotes non-normally distributed data (analyzed with Mann–Whitney *U* test).

### Within-group comparisons (pre- vs. post-intervention)

In the experimental group, all six COP parameters and the four clinical scale scores showed statistically significant improvement after the 6-week intervention compared to baseline (all *P* < 0.001, Table 3). Similarly, the control group also demonstrated significant improvements in all outcome measures post-intervention (all *P* < 0.001, Table 4).

**Table 3:** Within-group comparison of outcome measures in the experimental group (*n* = 30).

Variable	Baseline	Post-intervention	*T*/*Z*	*p*-value
Berg Balance Scale (score)*	25.00 (22.00,28.00)	35.00 (33.00,39.00.25)	−4.901	<0.001
Fugl-Meyer Assessment-LE (score)*	7.00 (6.00, 7.00)	16.00 (14.50, 17.00)	−4.854	<0.001
Timed Up and Go Test (s)*	19.00 (18.00, 21.00)	14.00 (13.75, 15.00)	−4.858	<0.001
Affected-Leg Stance Time (s)*	1.88 (0.889, 3.50)	4.00 (2.25, 5.53)	−4.739	<0.001
COP ML Sway Amplitude (mm)*	5.83 (4.49, 6.94)	3.43 (2.11, 4.38)	−4.773	<0.001
COP AP Sway Amplitude (mm)*	6.21 (5.00, 7.38)	3.60 (2.52, 4.45)	−4.783	<0.001
ML Sway Velocity (mm/s)*	8.08 (7.09, 10.09)	5.03 (3.90, 6.08)	−4.784	<0.001
AP Sway Velocity (mm/s)	11.42 ± 1.77	6.17 ± 1.16	33.920	<0.001
Motion length of COP in 30 (mm)	547.87 ± 69.93	255.32 ± 62.71	45.132	<0.001
Motion ellipse area (mm^2^)	552.15 ± 74.18	257.03 ± 62.44	38.510	<0.001

Data presented as mean ± SD or median (IQR).

* Wilcoxon signed-rank test used (non-normal distribution).

**Table 4:** Within-group comparison of outcome measures in the control group (*n* = 30).

Variable	Baseline	Post-treatment	*T*/*Z*	*p*-value
Berg Balance Scale (score)*	26.00 (22.00,28.00)	33.00 (29.00,35.50)	−4.878	<0.001
Fugl-Meyer Assessment-LE (score)*	7.00 (6.00, 8.00)	12.00 (11.00, 13.00)	−5.389	<0.001
Timed Up and Go Test (s)*	19.00 (17.00, 21.00)	16.00 (14.75, 18.00)	−5.031	<0.001
Affected-Leg Stance Time (s)*	2.10 (1.00, 3.00)	3.50 (2.00, 4.50)	−4.829	<0.001
COP ML Sway Amplitude (mm)	5.70 ± 1.32	3.90 ± 1.26	34.841	<0.001
COP AP Sway Amplitude (mm)	6.13 ± 1.25	4.18 ± 1.32	24.521	<0.001
ML Sway Velocity (mm/s)*	8.31 (6.47, 10.26)	6.20 (5.11, 7.56)	−4.781	<0.001
AP Sway Velocity (mm/s)*	11.26 (9.50, 13.59)	8.30 (6.73, 10.28)	−4.784	<0.001
Motion length of COP in 30 (mm)*	542.32 (489.60, 602.88)	355.38 (298.72, 406.59)	−4.770	<0.001
Motion ellipse area (mm^2^)	539.43 ± 76.61	377.80 ± 76.39	12.221	<0.001

Data presented as mean ± SD or median (IQR).

* Wilcoxon signed-rank test used (non-normal distribution).

### Between-group comparison of intervention efficacy

To compare the efficacy of the interventions while adjusting for baseline values, an ANCOVA was performed for each outcome measure. The experimental group exhibited significantly greater improvements than the control group across all four clinical scale scores and all six balance instrument parameters (all *P* < 0.001). The adjusted post-intervention means and the results of the between-group comparisons are presented in Table 5.

**Table 5:** Comparison of post-intervention outcome measures between the two groups adjusted for baseline using ANCOVA.

Variable (change in)	Baseline-post-treatment adjusted mean (95% CI)		*F* (1, 57)	*p*-value
	Experimental (*n* = 30)	Control (*n* = 30)		
Berg Balance Scale (score)	36.12 (35.41–36.83)	31.88 (31.17–32.59)	68.42	<0.001
Fugl-Meyer Assessment-LE (score)	15.89 (15.55–16.23)	12.11 (11.77–12.45)	285.64	<0.001
Timed Up and Go Test (s)	13.85 (13.57–14.13)	16.15 (15.87–16.43)	121.17	<0.001
Affected-Leg Stance Time (s)	4.53 (4.13–4.93)	3.01 (2.61–3.41)	27.86	<0.001
COP ML Sway Amplitude (mm)	3.27 (3.05–3.49)	4.06 (3.84–4.28)	24.32	<0.001
COP AP Sway Amplitude (mm)	3.44 (3.24–3.64)	4.34 (4.14–4.54)	38.74	<0.001
ML Sway Velocity (mm/s)	5.00 (4.78–5.22)	6.23 (6.01–6.45)	59.40	<0.001
AP Sway Velocity (mm/s)	6.30 (6.10–6.50)	8.17 (7.97–8.37)	158.64	<0.001
Motion length of COP in 30 (mm)	260.76 (251.64–269.88)	349.94 (340.82–359.06)	209.03	<0.001
Motion ellipse area (mm^2^)	264.88 (255.59–274.17)	369.95 (360.66–379.24)	255.05	<0.001

Adjusted means are estimated marginal means from the ANCOVA model, controlling for baseline values. Lower scores for sway parameters indicate better balance. CI, confidence interval.

### Safety data

All 60 enrolled participants completed the 6-week study protocol without any study-related adverse events. No incidents of falls, dizziness, or other discomfort requiring intervention discontinuation were reported or observed during the training sessions in either group.

## Discussion

The findings of this randomized controlled trial demonstrate that balance instrument training using the Pro-Kin system, as an adjunct to conventional rehabilitation, is superior to conventional rehabilitation alone in improving balance function and lower limb motor function in patients with ischemic stroke hemiplegia. Beyond statistical significance, the clinical relevance of these improvements is noteworthy. The mean improvement in BBS score in the experimental group (approximately 12 points) substantially exceeds the established minimal clinically important difference (MCID) of 5–6 points for patients with stroke (14, 15). Similarly, the improvement in TUGT (approximately 5 s) is likely clinically meaningful, aligning with or exceeding suggested MCID thresholds in similar populations (16). These findings suggest that the observed benefits are not only statistically robust but also translate into meaningful functional gains for patients.

Balance control involves both automatic lower-level processes and higher-level cortical regulation (17). Stroke-induced damage to higher centers disrupts this regulation, leading to neuromuscular abnormalities and balance dysfunction. Consequently, stroke survivors often exhibit asymmetric gait patterns, reduced speed, and impaired postural control, significantly increasing fall risk (18, 19). Traditional post-stroke rehabilitation can render patients passive recipients of treatment, heavily reliant on external aids (20). Hemiplegic gait not only alters gait parameters but also reduces walking efficiency, hindering overall rehabilitation (21).

Effective balance relies on the integration of visual, vestibular, and somatosensory inputs. Following a postural perturbation, these inputs are processed centrally to initiate appropriate corrective responses. Stroke often damages these sensory pathways, impairing balance and postural control. Physical training is a cornerstone of rehabilitation, promoting neuroplasticity to aid motor recovery (22). From a neuroanatomical perspective, repetitive task-specific training of the hemiplegic limb can strengthen synaptic connections and enhance neural plasticity (23).

Research indicates that removing visual feedback increases body sway and impairs balance adjustment (24). In hemiplegic stroke patients, central processing of sensory input is delayed, and abnormal muscle activation patterns (e.g., extensor synergy, poor ankle/knee control) further contribute to balance disorders (25). The Pro-Kin system addresses these issues by providing real-time visual feedback on COP movement. We hypothesize that this feedback allows patients to consciously adjust their posture, facilitating sensorimotor integration and potentially improving visuospatial perception (26). It is plausible that by training patients to control their center of gravity within a defined visual target area, the system enhances proprioceptive awareness and vestibular integration. Furthermore, the exercises likely promote coordinated contractions of the trunk and lower limb muscles, which are crucial for stable weight transfer and gait (27).While these proposed mechanisms are grounded in current theories of neuroplasticity and motor learning (22, 23), they remain speculative as our study did not include direct neurophysiological measures to confirm them. The observed improvements could be attributed to a combination of these factors, as well as increased patient engagement.

Beyond balance improvements, the experimental group demonstrated significantly greater gains in lower limb motor function, as evidenced by FMA-LE scores (median improvement 9 vs. 5 points, *P* < 0.001) and TUGT performance (median reduction 5 vs. 3 s, *P* < 0.001). These findings suggest that Pro-Kin training confers benefits that extend beyond static postural control to encompass dynamic, functional motor tasks. The observed improvements in lower limb motor function may be attributed to several mechanisms. First, the weight-shifting exercises inherent to Pro-Kin training require coordinated activation of hip, knee, and ankle musculature, thereby reinforcing motor unit recruitment and inter muscular coordination (28). Second, the real-time visual feedback may enhance motor learning by providing immediate knowledge of performance, facilitating error detection and correction—processes fundamental to motor skill acquisition (29). Third, the progressive nature of the training, with increasing difficulty based on individual performance, aligns with principles of neuroplasticity requiring challenging, task-specific practice (30). The significant improvements in all COP parameters, particularly sway velocity and path length, indicate enhanced automatic postural responses that likely contribute to more efficient and safer gait patterns.The significantly greater improvement in affected-side single-leg stance time (TALS) in the experimental group (median improvement 2.0 vs. 1.0 s, *P* < 0.001) is particularly noteworthy, as single-leg stance is a challenging task requiring integrated sensorimotor control and is strongly associated with fall risk in stroke populations (31). This finding suggests that Pro-Kin training effectively targets the specific deficits limiting single-limb support during gait. Furthermore, the superior reduction in mediolateral sway parameters (ML amplitude and velocity) observed in the experimental group indicates improved frontal plane stability, which is crucial for weight transfer during walking and reducing falls risk (32).

The structured, game-based nature of the Pro-Kin training may also increase patient motivation and engagement compared to routine exercises, potentially leading to better adherence and outcomes. Additionally, this technology-assisted method can reduce the physical burden on therapists. It is also important to acknowledge a potential limitation related to the study design. While the outcome assessor was blinded, the participants and treating therapists were not, due to the visible nature of the technology-assisted intervention. The engaging, game-based nature of the Pro-Kin training could have introduced motivational and expectancy effects, potentially influencing the outcomes in the experimental group. This performance bias is an inherent challenge in non-pharmacological intervention studies and should be considered when interpreting the results.

This study has several limitations. First, the sample size was relatively small (*n* = 60), and all participants were recruited from a single center, which may limit the generalizability of the findings. Furthermore, the inclusion criteria required patients to have a certain level of standing balance (standing balance function rated at level 1 or higher and MRC grade ≥3), meaning our findings are primarily applicable to subacute and convalescent patients with moderate-to-mild impairment and may not be generalizable to the broader, more severe spectrum of the stroke population. Future research should involve larger, multicenter samples, including patients with varying severity levels. Second, the absence of long-term follow-up assessments prevents determination of whether the observed benefits are sustained beyond the 6-week intervention period. Future research should therefore include extended follow-up periods (e.g., 3 and 6 months post-intervention) to evaluate the durability of treatment effects and their impact on clinically meaningful outcomes such as fall incidence and community ambulation. Third, multiple outcome variables were tested simultaneously without statistical correction for multiple comparisons, which increases the risk of a Type I error. Although the highly consistent and significant improvements across all measures (all *P* < 0.001) mitigate this concern to some extent, future confirmatory studies should either pre-specify a primary outcome or apply appropriate corrections, such as Bonferroni adjustment.

## Conclusion

Balance instrument training using the Pro-Kin system is an effective intervention for improving both balance function and lower limb motor function in this specific cohort of patients with ischemic stroke hemiplegia, and in this short-term context, it demonstrated superior efficacy compared to conventional rehabilitation training alone across all clinical and instrument-derived outcomes. This approach not only enhances static and dynamic postural stability but also improves functional mobility and motor recovery of the affected lower limb, while offering a potentially more engaging and less labor-intensive rehabilitation modality for both patients and therapists.

## Data Availability

The original contributions presented in the study are included in the article/Supplementary Material, further inquiries can be directed to the corresponding authors.
